# Applications of Ionic Liquids in Whole-Cell and Isolated Enzyme Biocatalysis

**DOI:** 10.3390/molecules26164791

**Published:** 2021-08-07

**Authors:** Hasan Tanvir Imam, Vladimír Krasňan, Martin Rebroš, Andrew Craig Marr

**Affiliations:** 1 School of Chemistry and Chemical Engineering, Queen’s University Belfast, David Keir Building, Stranmillis Road, Belfast BT9 5AG, UK; H.Imam@qub.ac.uk; 2 Institute of Biotechnology, Faculty of Chemical and Food Technology, Slovak University of Technology in Bratislava, Radlinského 9, 812 37 Bratislava, Slovakia; vladimir.krasnan@stuba.sk

**Keywords:** Ionic liquids, biocatalysis, whole-cell biocatalysis, isolated enzyme biocatalysis

## Abstract

Ionic liquids have unique chemical properties that have fascinated scientists in many fields. The effects of adding ionic liquids to biocatalysts are many and varied. The uses of ionic liquids in biocatalysis include improved separations and phase behaviour, reduction in toxicity, and stabilization of protein structures. As the ionic liquid state of the art has progressed, concepts of what can be achieved in biocatalysis using ionic liquids have evolved and more beneficial effects have been discovered. In this review ionic liquids for whole-cell and isolated enzyme biocatalysis will be discussed with an emphasis on the latest developments, and a look to the future.

## 1. Introduction

**Figure 1:**
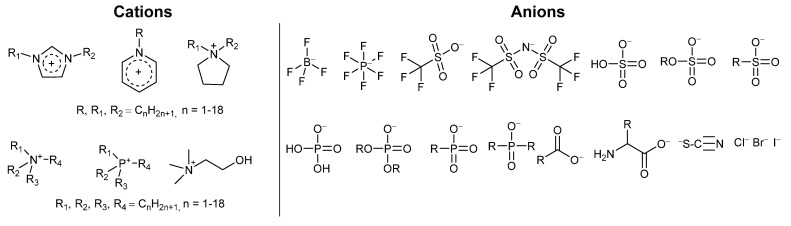
Common ionic liquids (cations and anions) used in biocatalysis applications.

Ionic liquids comprise solely of cations and anions. They are liquid under operating conditions as they do not pack together as closely as conventional ionic compounds such as sodium chloride. This can be rationalized due to the irregular shapes of the ions. Some common classes of ion that have been applied to biocatalysis are given in Figure 1.

Examples of ions employed in ionic liquids used for biocatalysis include: **Cations:** Alkyl imidazolium, e.g., 1,3-dimethylimidazolium [MMIM], 1-ethyl-3-methylimidazolium [EMIM], 1-butyl-3-methyl-imidazolium [BMIM], 1,3-butylimidazolium [BBIM], 1-hexyl-3-methyl-imidazolium [HMIM], 1-octyl-3-methyl-imidazolium [OMIM]; Alkyl pyridinium, e.g., hexyl-pyridinium [HPYR]; Alkyl pyrrolidinium, e.g., 1-butyl-1-methylpyrrolidinium [BMP], 1-hexyl-1-methylpyrrolidinium [HMP]; Alkylammonium, e.g., tetramethylammonium [N_1,1,1,1_], triethylmethylammonium [N_2,2,2,1_], butyltrimethylammonium [N_4,1,1,1_], trioctylmethylammonium [N_8,8,8,1_]; Alkyl phosphonium, e.g., Ethyl-tributyl phosphonium [P_2,4,4,4_], Trihexyltetradecylphosphonium [P_6,6,6,14_], Tritetradecylhexylphosphonium [P_14,14,14,6_]; Cholinium [Ch] **Anions:** tetrafluoroborate [BF_4_]; hexafluorophosphate [PF_6_]; tri-fluoromethanesulfonate (OTf); bis[(trifluoromethyl)sulfonyl]amide [NTf_2_]; hydrogen sulphate [HSO_4_]; alkyl sulphates (e.g., [EtSO_4_]); alkyl sulfonates (e.g., [C_8_SO_3_]); dihydrogen phosphate (dhp) [H_2_PO_4_]; dialkyl phosphates (e.g., [Et_2_PO_4_]); alkyl phosphonates (e.g., [MeO(H)PO_2_]); bis(2,4,4-trimethylpentyl)phosphinate [Phosp]; acetate (OAc) [CH_3_COO-]; butyrate [CH_3_CH_2_CH_2_COO-]; amino acids (e.g., glutamate); thiocyanate [SCN]; chloride [Cl]; bromide [Br]; iodide [I].

The presence of organic groups and heteroatoms within the ions gives rise to non-ionic interactions such as hydrogen bonding and Van der Waals forces which help to determine physical properties such as viscosity, hydrophilicity, and glass transition temperature. Altering the groups within the ions enables chemists to vary these physical properties systematically. In general, ionic liquids behave as polar non-volatile solvents with a wide solvent range.

**Figure 2:**
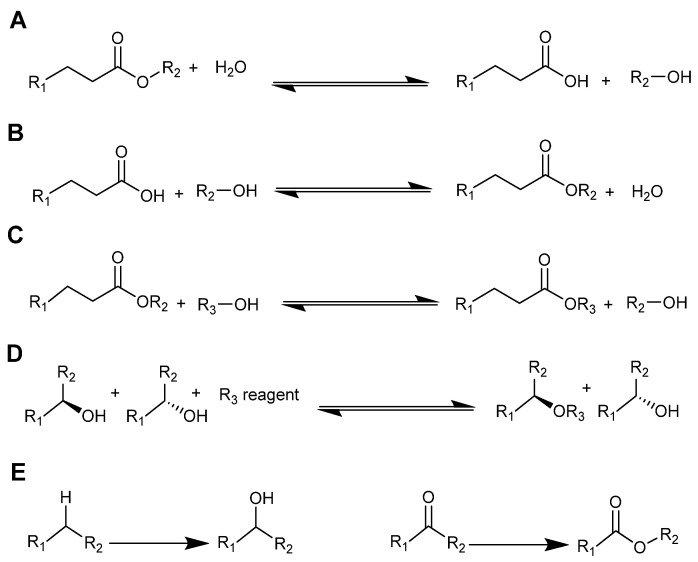
Reaction schemes of some common biocatalytic processes studied in ionic liquids. (**A**) Hydrolysis, (**B**) esterification, (**C**) transesterification, (**D**) kinetic resolution, and (**E**) oxidation.

Biocatalysis is a rapidly expanding area of scientific research in academia and industry. Interest has been driven by the many uses of enzymes, and the low environmental impact of biocatalysts and biocatalytic processes. Biocatalysts operate at low temperatures and catalyse reactions cleanly with high selectivity [1]. Some common types of enzymatic catalytic process in ionic liquids have been given in Figure 2.

**Figure 3:**
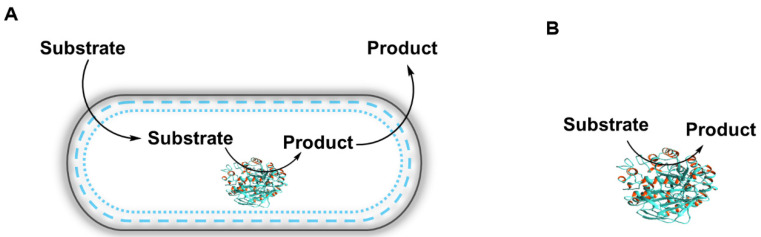
Schematic representations of biocatalytic processes. (**A**) Whole-cell biocatalysis and (**B**) isolated enzyme biocatalysis.

Applied biocatalysis can be divided into two disciplines (Figure 3). The first is whole-cell biocatalysis. This class employs whole microorganisms such as bacteria and yeasts and is commonly applied to food processing and chemical synthesis, such as the synthesis of 1,3-propanediol by DuPont Tate & Lyle Bio Products. The second class is the use of isolated enzymes, whereby the catalytic protein has been removed from the cell, this is commonly applied within detergents and for fine chemical synthesis. Pure enzymes are usually used when high purity is desired with no side reactions, whole-cell compartments, or metabolite cross-contamination. This method requires simpler technical equipment (often with no pH, O_2_ control). Whole-cell biocatalysis is applied when lower production costs are desired (the enzyme downstream processing cost is eliminated), when the addition of enzyme cofactors or their recycling is required, or in case of cascade reactions (when more than one enzyme is used).

The application of ionic liquids within the two classes is very different, and they are best considered separately.

## 2. Ionic Liquids and Whole-Cell Biocatalysis

### 2.1. Introduction to Whole-Cell Biocatalysis

In whole-cell biocatalysis, the reaction is performed by an enzyme as part of a microorganism such as a bacterium or yeast. An enzyme operating within a whole-cell biocatalyst is protected by the environment of the cell.

Compared to crude, purified, or immobilized isolated enzymes, this approach has several important advantages. It is the cheapest method in terms of biocatalyst preparation, especially due to the significant reduction of downstream processes [2]. If cofactors are needed in redox reactions, such as oxidations, many whole-cell biocatalysts are able to provide and regenerate them, which significantly reduces the production cost [3]. Furthermore, cell membranes can act as protection against harsh conditions resulting in increased enzymes stabilities [4]. Thanks to this, it is possible to apply various non-traditional reaction environments such as micro-aqueous solvent systems [5] or reactions in ionic liquids [6].

Whole-cell biocatalysis has developed considerably over the last decades and has been implemented in many industrial processes producing, for example, enantiopure chemicals. Thus, currently 75% of the processes of industrially implemented redox biotransformations use whole cells [7,8]. One example is the chemoenzymatic synthesis of (*S*)-rivastigmine—a drug used to combat Alzheimer’s type dementia. Biocatalytic reduction of 3-acetylphenyl-*N*-ethyl-*N*-methylcarbamate to the corresponding secondary alcohol was achieved by whole cells of *Lactobacillus reuteri* DSM 20016. The biocatalytic step reached 90% yield, and up to 98% ee purity. In addition, the process has the potential for large-scale production due to the simple and cheap preparation of *L. reuteri* cells, easy-to-make subsequent chemical steps, and the low environmental impact of the process [9]. Another example of the application of whole-cell biocatalysis is the production of 1,3-propandiol, a monomer used in the synthesis of polymers including polytrimethylene terephthalate. DuPont and Genecor genetically modified the *E. coli* K12 strain to optimize production of 1,3-propanediol from glucose. The final mutant strain was successfully applied in a DuPont Tate and Lyle Bio products plant with a capacity of over 63,000 t per year. 1,3-propanediol is prepared from glucose derived from a corn starch feed [10]. The overall process requires a number of separation and purification steps in order to isolate the product from the aqueous fermentation broth and by-products, but nevertheless the overall process has a significantly lower carbon footprint than petrochemical routes.

The examples above illustrate the wide breadth of applications possible for modern whole-cell biocatalysis, with successful chemical synthesis in industry going from large-scale bulk chemicals down to the smaller scale pharmaceutical and fine chemicals. Whole-cell biocatalysis has a particular advantage when cofactors are required for the reaction, for example in some oxidations and reductions, or when the protein is particularly fragile once removed from the cell.

As summarized by Solano et al. in 2012, twenty compounds have been reported to be produced by companies using whole-cell biocatalysts [11]. Whole cells are involved in the preparation of drugs such as atorvastin, antitumoral agents and HIV endopeptidase inhibitors, and the anti-asthma drug Montelukast.

It must be added that biotransformations with whole cells have several drawbacks, which limit their application. One of the problems is the stability of the catalyst, which can be negatively affected by substrate or product inhibition, high temperature, extreme pH, or by organic solvents. Improvement can often be achieved by immobilizing the biocatalyst. For example, immobilization within hydrogels e.g., polyvinyl alcohol (PVA) particles can increase pH and temperature tolerance, and enable biocatalyst recyclability and recovery. In addition, immobilization can increase the storage stability of the biocatalyst, allow the application of a non-aqueous reaction medium, or enable the use of substrates that are toxic to free cells [12,13]. Another disadvantage linked with whole-cell biocatalysis is the formation of by-products by cell metabolism which may result in biocatalyst inhibition or complicate the downstream processing of the final product. This could be overcome by selective inactivation of cell metabolism, leaving only the essential pathway through metabolic engineering [2,14]. Complications can also be caused by cell membranes and their limited permeability. This can cause limitations in transport and greatly reduce enzymatic efficiency. The most common solution is permeabilization of cells by specific detergents, solvents, or heat shock [15]. More sophisticated methods, with lower cell stress, include fluidity modification by membrane fatty acids modification and genetic engineering of membrane transporters. Relocation of the target biocatalyst into the periplasm can also be applied, increasing access of the substrate to the target enzyme, [4,16].

### 2.2. Ionic Liquids as Additives in Whole-Cell Biocatalytic Processes

An important factor limiting the application of whole-cell biocatalysis is low tolerance to organic solvents. The highest biocatalytic activity is achieved in the natural environment, and this is usually mimicked using aqueous based buffers. This approach limits the use of many substrates and the synthesis of hydrophobic products due to poor water solubility. One of the strategies to solve this problem is to create biphasic systems, where the biocatalyst is in the aqueous phase and the substrate/product is in the organic phase [17]. However, it is often difficult to find a biocompatible solvent and the environmental toxicity of organic reagents must be considered. A feasible alternative to organic solvents is provided by ionic liquids [6] which are much safer to use than many organic solvents and bring the possibility of designing a bespoke tailored solvent. An early attempt at organic solvent replacement was reported by Lye and co-workers using an ionic liquid containing the PF_6_ anion ([BMIM][PF_6_]) to confer hydrophobicity [18]. The attempt was successful, and 1,3-dicyanobenzene was transformed into erythromycin-A by *Rhodococcus R312* and subsequently extracted. At this time the variety of ionic liquids available was limited. [BMIM][PF_6_] would not be considered nowadays due to concern over the stability of the perfluorinated anion.

To date, ILs have found applications in a variety of whole-cell biocatalytic processes. ILs usually act as the non-aqueous phase in a two-phase system, allowing the biocatalyst to remain in the water phase. Diffusion of compounds between the two phases is an important parameter in order to reach sufficient process performance parameters such as conversion and yield [19]. Despite the high biocompatibilies reported in many publications focused on ILs in whole-cell biocatalysis, a certain degree of toxicity and biological activity may be observed [20]. Therefore, the application of ILs in biocatalysis is usually a compromise between two factors: (1) solvent properties that have a positive impact on the desired chemical reaction/separation, and (2) toxic effects of solvents on cell metabolism [21]. Despite this, IL applications in multi-phase systems represent a significant improvement of biocatalysis due to increases in substrate solubility, and often offer improvements in product isolation. Low toxicity, recyclability, and the possibility to tune the solvent for the specific reaction could lead to the replacement of traditional organic solvents by ILs on the industrial scale in the near future.

As an example, *E. coli* bacteria, with a wide range of confirmed applications, are able to tolerate a large number of ILs, with low inhibition effects. Wood et al. in 2011 [22] tested over ninety ILs, of which low toxicity was exhibited by imidazolium salts with short alkyl chains when paired with alkyl sulphate anions. ILs with longer alkyl chains may penetrate into the cell layer and disturb its structure.

### 2.3. Literature on the Effects of Ionic Liquids on Whole-Cell Biocatalysis

An extensive comparison of the toxicity of ionic liquids to different microorganisms was reported in the review of Egorova and Ananikov in 2018 [19]. According to the data reviewed, cholinium ILs and imidazolium ILs seemed to be the best options for whole-cell biocatalysis. In addition, the effect of alkyl chains was confirmed. In the case of organism resistance, the *Penicillium* genus showed the highest IL tolerance, whereas *S. cerevisiae* manifested high sensitivity to most ILs. Cholinium ILs are of particular interest as this cation is derived from a natural product and has the potential to overcome toxicity and cost concerns.

Liu et al. demonstrated the application of hydrophobic phosphonium ionic liquids [23]. The *C. butyricum* bacterium, which can be used to produce 1,3-propanediol from glycerol, was shown to tolerate ILs that can extract the target product. Although, IL [P_6,6,6,14_][C_8_SO_3_] affected cell growth, production of 1,3-propenediol was shown to be unaffected or increased. Maximal growth rates were more than 50% compared to the control, even in a fermentation medium that was 1:1 aqueous: IL. The ionic liquid containing 1,3-propanediol was used in subsequent homogeneous chemocatalyst promoted chemical reactions. The overall final process represented a combined bio-chemo-catalysis of waste glycerol to valuable products [24,25,26]. This phosphonium sulfonate IL was shown to have very low water solubility and good biocompatibility, essential attributes for a second solvent in whole-cell biocatalysis.

The preparations of many key pharmaceutical intermediates (often by recombinant *E. coli*) were enhanced using different ILs, examples of which have been summarized in published reviews [6,19,22]. Reports include IL-assisted enantioselective reduction of ketones, biofuel production, oxidation, hydrolysis, transesterification, and nucleoside acylation. The influence of ionic liquids on whole-cell biocatalysis was described in book chapters [27,28]. Further examples of the application of ILs in whole-cell biocatalysis reported between 2015 and 2021 are given in Table 1. This body of previous work confirms the positive impact of ILs on whole-cell biocatalytic reactions.

In many cases, ILs not only serve to create a reaction medium and limit the toxic effect of substrates/products, but also directly influence the reactions themselves by interacting with the biocatalyst or other reaction intermediates. In the coming years, the gradual implementation of these liquids into production processes can be expected. This will be assisted by the increased number and variety of ionic liquids available. Recent efforts have begun to concentrate on ionic liquids with lower environmental impact and comprising simple bioderived ions such as cholinium and amino acids.

**Table 1:** Examples of the application of ionic liquids within whole-cell biocatalytic reactions.

Reaction	IL System	MO	Ref
Helicid + vinyl benzoate → 6′-*O*-benzoyl-helicid	Acetone/[BMIM][PF_6_] (5%, *v*/*v*)	*Aspergillus oryzae*	[29]
*o*-chloromandelonitrile → (*R*)-*o*-chloromandelic acid	[BMIM][PF_6_]-based biphasic system	*Escherichia coli* BL21 (DE3) nitrilase mutant F189T/T132A	[30]
caffeic acid + 2-phenyl ethanol → caffeic acid phenethyl ester	[EMIM][NTf_2_]	*Aspergillus niger* EXF 4321	[31]
Phytosterols → Androst-4-ene-3,17-dione	1% (*v*/*v*) [Ch][Asp]	*Mycobacterium* sp. MB 3683	[32]
3,5-bis(trifluoromethyl) acetophenone → (*R*)-[3,5-bis(trifluoromethyl)phenyl] ethanol	[N_1,1,1,1_][PF_6_] + distilled water reaction system	*Trichoderma asperellum* ZJPH0810	[33]
benzaldehyde + glucose → (*R*)-phenylacetylcarbinol	[BMIM][PF_6_]-based biphasic system	*Saccharomyces cerevisiae* BY4741	[34]
(4-chlorophenyl)-(pyridin-2-yl) methanone → (*S*)-(4-chlorophenyl)-(pyridin-2-yl) methanol	[EMIM][(MeO)HPO_2_]	*Cryptococcus* sp. M9-3	[35]
Geraniol → geranyl glucoside	[HPYR][NTf_2_]	*Escherichia coli* expressing VvGT14a	[36]
(*R*)-carvone → (2*R*,5*R*)-dihydrocarvone	20% (*v*/*v*) [HMP][NTf_2_]	*Escherichia coli* overexpressing ene-reductase	[37]
*N*-ethyl-methyl-carbamic acid → *N*-ethyl-methyl-carbamic acid-3-[(1*S*)-hydroxy-ethyl]-phenyl ester	[BMIM][BF_4_]	*Escherichia coli* BL21 (DE3)	[38]

## 3. Ionic Liquids and Isolated Enzymes

Many enzymes can be removed from the parent cell and used as purified or partly purified proteins. When the protein responsible for enzyme activity is removed from the cell, it is vulnerable to the chemical environment ‘on the outside’. This can lead to poisoning, denaturing and unfolding of the protein, and ultimately a loss of activity. The main application of ionic liquids to isolated enzyme biocatalysis is to assist the stabilization of the protein; while performing this task, ionic liquids can also help to facilitate separations.

As ionic liquids have a number of highly variable properties, it does not serve scientists well to form generalizations about the effects of ionic liquids upon proteins. A large variety of enzymes and ionic liquids are available, thus offering many opportunities to manipulate an enzyme-IL pair to maximize enzyme solubility and stability. General properties of ionic liquids that render them potentially useful in isolated enzyme biocatalysis are low volatility, high solvating ability, and relatively slow diffusion. Ionic liquids have been observed to stabilize proteins in many studies. Useful information on enzyme stability and biocatalysis in ionic liquid media can also be found in recent reviews [39,40,41,42,43,44,45,46,47,48,49].

**Figure 4:**
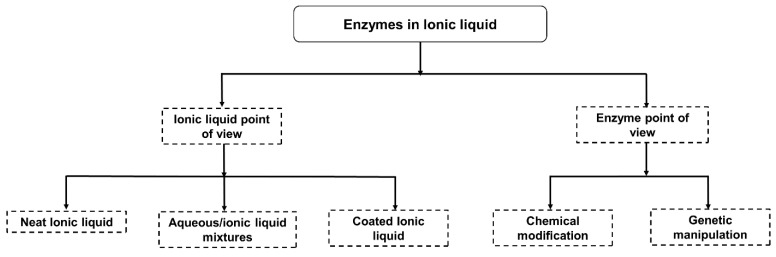
Different approaches to studying enzymes in ionic liquids.

Modern research into maximizing isolated enzyme solubility, stability, and activity in ILs can be approached in two ways (i) IL-based technologies which can include manipulation of enzymes in neat IL, aqueous/hydrated ionic liquids and coating in ILs. (ii) Enzyme-based technologies that include modifying the polarity (hydrophobicity, dielectric constant) of the enzyme by covalent modification, and genetic manipulation by site-directed mutagenesis of amino acids (Figure 4).

### 3.1. Studying Enzymes in Ionic Liquids: Spectroscopic and Analytical Methods

Most of the enzyme structure-activity information available in the protein data bank (www.rcsb.org accessed on 1 August 2021) or bioinformatics tools (www.expasy.org accessed on 1 August 2021) has been determined in aqueous media [50,51]. Enzyme structural stability in neat organic solvents or aqueous/organic biphasic media is commonly measured by activity assay. The physical and chemical properties vary between ionic liquids and can differ significantly from aqueous and common organic solvents, thus studying enzyme’s structure–activity relationships in ionic liquids is a challenging and rewarding research topic. In addition to assays of enzyme activity, there are several analytical methods that can be used to glean information on enzymes in ionic liquids. However, the spectroscopic and analytical investigation of an enzyme’s biophysical and structural properties in ionic liquids is largely hampered by relatively high viscosity and the low solubility of biomolecules in common ionic liquid media. In addition, spectroscopic/analytical instruments and data acquisition protocols are designed for aqueous and organic solvents, and thus special procedures are required to study proteins in ionic liquids. Notwithstanding, many research works have been carried out to decipher the structural properties of enzymes in ionic liquid media using conventional structural and bioanalytical tools. In this section some exemplar studies have been highlighted that employ spectroscopic methods to probe the ionic liquid–protein interaction. Many of the oldest and most established ionic liquids, such as 1-alkyl-3-methylimidazolium halides, have well documented negative effects on proteins. In later sections complementary enzyme–ionic liquid interactions will be discussed.

X-ray crystallography has been employed to crystalize a lysozyme enzyme in the presence of [BBIM][Cl] as an additive at different concentrations (0.1–0.4 mol/L) [52]. The structural analysis suggested that the ionic liquid cation bound to the protein via two tryptophan residues (W62 and W63) and one aspartate residue (D101) without changing the enzyme conformation. In addition, the ionic liquid was able to crystalize lysozymes from impure natural samples suggesting a separation and purification role.

Lysozyme was crystallized with 1.2 Å resolution in the presence of 1 mol% protic ethylammonium nitrate (EAN) ionic liquid–water mixture [53]. The crystal structure revealed that the nitrate (NO_3_) anions formed hydrogen bonds with the surface water molecules and interacted with lysozyme’s surface residues of arginine (R14), histidine (H15), aspartate (D18), serine (S24), glutamine (Q 121), and asparagine (N65, N74, N77) without a significant change to the protein secondary structure.

Ionic liquid [BMIM][Cl] (5–20% *v*/*v*) was used to obtain crystal structures (1.2–1.9 Å) of lipase A from *Bacillus subtilis*. Nordwald et al. suggested that, at 20% *v*/*v*, the ionic liquid destabilized the enzyme by interacting with tyrosine (Y49) via a cation-π interaction, and with glycine (G158) via a hydrophobic interaction [54]. Mutation to glutamic (E) acid of Y49E and G158E reduced the interaction and stabilized the enzymes. This demonstrated that enzyme surface charge modification could be an option for stabilizing enzymes in ionic liquids.

High-resolution Magic Angle Spinning NMR (HR-MAS-NMR) spectroscopy has been used to study the structure and conformation properties of model protein GB1 in aqueous [BMIM][Br] [55]. The method was able to highlight protein–IL interactions at IL concentrations ranging from 0.6–3.5 mM (10–60% *v*/*v*). [BMIM][Br] interacted strongly with the α-helix of the protein. At 50% *v/v* the protein secondary structure remained almost unchanged, however, both folded and unfolded protein structures were observed at 60% *v/v* IL. The folding–unfolding event was reversible.

Human serum albumin (HSA) stability in imidazolium based ionic liquids with chloride ions was found to depend on the alkyl chain length as studied by saturation-transfer difference STD-NMR and ^35^Cl NMR spectroscopy [56]. The longer the chain length, the stronger the hydrophobic interaction with the protein surface, leading to replacement of the structural water, and resulting in structural instability. Modifying the imidazolium alkyl chain by appending hydroxyl or methoxy groups stabilized HSA. ^19^F-NMR spectroscopy was used to study the stability of a fluorine labelled KH1 domain and the N-terminal SH3 domain of drk (SH3) in [BMIM][Br] [57].

Circular dichroism (CD), fluorescence, infrared, and Raman spectroscopy are widely used to probe enzyme secondary protein structure in ionic liquids. Circular dichroism (CD) and molecular dynamics (MD) methods were used to study the inactivation of the endocellulase 1 (E1) from *Acidothermus cellulolyticus* in 0–20% *w/v* [BMIM][Cl] [58]. The IL interacted most significantly with the α-helix and this led to deactivation. This happened in two steps; firstly, rapid reversible binding followed by slow irreversible deactivation. Fluorescence and CD spectroscopic studies on the stability of proteolytic enzyme stem bromelain from *Ananas comosus* in 0.01–1 M IL of 1-alkyl-3-methylimidazolium chlorides suggested that the protein remained stable at low IL concentrations of 0.01–0.1 M. Higher concentrations of IL destabilized the protein [59]. The protein stability was dependent on the alkyl chain length. Higher alkyl chains interacted more strongly with the protein and resulted in protein aggregation. The secondary structures of lysozyme, trypsin, β-lactoglobulin, and α-amylase have been investigated in protic ILs by using infrared (IR) spectroscopy [60].

A combined approach of using different spectroscopic techniques including circular dichroism (CD), fluorescence, ultraviolet-visible (UV/Vis) and nuclear magnetic resonance (NMR) spectroscopies, and small-angle X-ray scattering (SAXS) spectroscopy studies was used to study Green Fluorescent protein in ILs of [BMP] and [BMIM] cations with [OAc-], [Cl-], and [OTf-] anions. The data suggested that all the ILs screened reduced the thermal stability and impacted the secondary and tertiary structures of the protein [61]. The anions exerted more pronounced effects on the structure than the cations.

### 3.2. Enzyme Activity in Neat Ionic Liquids

Dissolving enzymes in neat ILs can be difficult, with dissolution taking days at elevated temperatures of 30–60 °C [62,63,64]. The requirement for elevated temperatures and long times suggests significant changes in inter- and intramolecular charge–charge interactions are required.

*Candida antarctica* lipase B (CALB) was dissolved in neat hydrophilic ILs at 40 °C over 24 h; however, the enzyme was found to be insoluble in a hydrophobic IL [62]. Despite being soluble in IL, CALB exhibited low activity in the transesterification of ethyl butanoate with 1-butanol. The enzyme retained 26% activity in [N_2,2,2,1_][MeSO_4_], while in [EMIM][EtSO_4_] and [BMIM][NO_3_] only 7% and 3% activity were observed, respectively. The reduction in activity arose from the change in the enzyme secondary structure as a result of strong hydrogen bonding of the anions with the protein. Interestingly, the enzyme recovered from the [BMIM][NO_3_] IL retained 73% activity. This suggests that a lack of hydration may have contributed towards the lower activity in the neat IL and the enzyme refolded with hydration. This underlines an important principle, in order to preserve the natural structure and activity of the enzyme, the hydration of the protein must be preserved. Consequently, ions that diffuse relatively quickly and displace water from around the protein are not expected to support good enzyme activity.

Horse heart Cytochrome C (CytC) dissolved in neat IL [EMIM][EtSO_4_] exhibited three times higher peroxidase activity than in aqueous phosphate buffer [63]. UV-Visible and CD spectroscopy revealed that a possible distortion of the Fe-S(Met80) bond of the Fe-heme group may have contributed towards the higher peroxidase activity. This suggests that neat ILs can stabilize unusual enzyme conformations and bring about unprecedented activity.

Lysozyme was solubilized in neat [EMIM][EtSO_4_], [P_2,4,4,4_][Et_2_PO_4_] and [BMIM][SCN] and shown to exhibit different stabilities in different ILs [64]. Lysozyme completely unfolded in [BMIM][SCN], but retained secondary structure in [EMIM][EtSO_4_] and [P_2,4,4,4_][Et_2_PO_4_] at room temperature. [P_2,4,4,4_][Et_2_PO_4_] afforded the enzyme good thermal stability, while the enzyme in [EMIM][EtSO_4_] was susceptible to unfolding due to thermal hydrolysis. The strong hydrogen bond acceptor ability of the SCN anion led to strong interactions with the enzyme amide groups, resulting in a disrupted protein structure. The [EMIM] cation is a strong hydrogen bond donor and can interact with the carboxyl groups on the protein, whereas [P_2,4,4,4_] has no hydrogen bond donor ability.

The activity of *Candida antarctica* lipase B (CALB) in catalysing kinetic resolution of (*R,S*)-1-(1-naphthyl)ethylamine in [HMIM][NTf_2_] was comparable to that in toluene [65]. Increasing the alkyl chain from hexyl to octyl and decyl decreased the activity.

**Figure 5:**
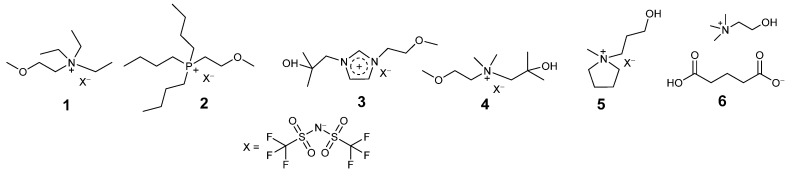
Representative examples (**1**–**6**) of functionalized ionic liquids for enzyme studies.

*Candida antarctica* lipase B (CALB) catalysed the non-aqueous transesterification of ethyl sorbate and 1-propanol and ring-opening polymerization (ROP) of ε-caprolactone in ether functionalized ionic liquids of different cations of imidazolium, phosphonium, alkyl ammonium, piperidinium, and pyridinium with [NTf_2_] [66]. Better transesterification and ring polymerization activity were observed in [CH_3_OCH_2_CH_2_-Et_3_N][NTf_2_] (**1**, Figure 5) and [CH_3_OCH_2_CH_2_-PBu_3_][NTf_2_] (**2**, Figure 5) ionic liquids respectively, compared to non-ether functionalized and other ether functionalized ILs. *Candida antarctica* lipase B (CALB) exhibited 2- and 1.5-times higher transesterification activity of ethyl sorbate and 1-propanol in dual ether and tert-butanol functionalized ILs of imidazolium [CH_3_OCH_2_CH_2_-IM-*t*-BuOH][NTf_2_] (**3**, Figure 5) and ammonium [CH_3_OCH_2_CH_2_-Me_2_N-*t*-BuOH][NTf_2_] (**4**, Figure 5) respectively, compared to organic solvent t-BuOH [67].

### 3.3. Enzymes in Aqueous/Ionic Liquid Mixtures

The solubility of proteins in neat ionic liquids can be poor. In addition, the high viscosity of neat ionic liquids can reduce enzyme activity due to substrate mass transfer limitations. Dissolution can be improved, and this challenge circumvented, by dissolving the enzyme in hydrated or aqueous ionic liquid. Solvent mixtures vary from ionic liquids diluted with water to aqueous buffers modified by the addition of small concentrations of IL.

#### 3.3.1. Enzymes in Aqueous/Ionic Liquid Mixtures with at Least 50% IL

In this section work is highlighted in which isolated enzymes have been utilized in aqueous/ionic liquid mixtures in which the IL is predominant.

*Candida antarctica* lipase B (CALB) in 2% aqueous [EMIM][NTf_2_] and [N_4,1,1,1_][NTf_2_] exhibited 3.7- and 2.0-times higher activity in the esterification of butyl butyrate than in hexane [68]. The authors postulated that these hydrophobic ILs formed a microenvironment around the enzyme.

Cellobiose dehydrogenase (CDH) activity was compared in 20 and 35% aqueous [Ch][dhp]. The enzyme was inactive for oxidation at 20% water, however, at 35%, the enzyme oxidized cellobiose [69]. The difference in activity was attributed to the different viscosities of the mixtures.

Concanavalin A (ConA) remained well folded, and retained its sugar binding ability in 20% aqueous [Ch][dhp], while in [BMIM][BF_4_] or hydrated [EMIM][MeO(H)PO_2_], the enzyme lost both structure and sugar binding ability [70]. IL component ions and the degree of hydration influenced the solubility and stability of the enzymes. Increasing the water content decreased the stability.

Epidermal growth factor receptor monoclonal antibody (EGFR mAb) remained structurally stable and showed better protection against protease degradation in 50% hydrated [Ch][dhp] IL compared to EGFR mAb in PBS buffer [71]. Higher IL concentrations gave better stability and antigen binding but resulted in higher aggregation.

Lipase was dissolved in hydroxyl-functionalized pyrrolidinium based IL (**5**, Figure 5) and applied to transesterification of soybean oil and methanol for biodiesel production in 28.6% aqueous IL [72]. The enzyme was easily separable from the IL simply by water and acetone wash; however, the recovered enzyme lost some activity.

The enzyme Cytochrome C (CytC) retained its structure and catalytic activity in choline based IL (**6**, Figure 5) [73]. In 50% aqueous IL 6, the enzyme exhibited 50-times higher activity for 2,2′-azino-bis(3-ethylbenzothiazoline-6-sulfonic acid) (ABTS) oxidation in the presence of H_2_O_2_ compared to the enzyme in aqueous buffer. This good result highlights the potential of cholinium ionic liquids in isolated enzyme biocatalysis.

Horse heart Cytochrome C (CytC) exhibited 5 times higher peroxidase activity for guaiacol oxidation to tetraguaiacol in ionic liquid microemulsions containing biamphiphilic IL 1-hexyl-3-methylimidazolium dioctylsulfossuccinate [HMIM][AOT] (65% *w/w*), hydrophobic [EMIM][NTf_2_] (29% *w/w*), and buffer (6% *w/w*) [74]. The enzyme showed better storage stability in the microemulsion for 10 days compared to the buffer.

Apart from solubilizing, stabilizing, and enhancing enzyme activity, hydrated/aqueous ILs can play a role in enzyme refolding and thermal stability.

Horse heart Cytochrome C (CytC) was dissolved in 20% aqueous [BMP][dhp] and [Ch][dhp], and the enzyme was found to exhibit enhanced thermal stability up to 110–130 °C [75]. The water content was found to play a significant role in the thermal stability. Increasing the water content to 80% drastically decreased the CytC thermal stability, and the denaturation temperature was found to be 77 and 62 °C, respectively; temperatures comparable to the denaturation temperature in water. At higher water content, the IL behaved as a solute and the individual cations and anions could diffuse rapidly and interacted freely with the enzyme, resulting in enzyme inactivation.

Lysozymes and recombinant human interleukin-2 were dissolved in various concentrations of 20–50% hydrated [Ch][dhp]. Increasing the IL content provided better thermal stability. Heating the IL-enzyme above the enzyme melting temperature (Tm) and subsequent cooling, resulted in protein misfolding and aggregation. At an IL concentration of 80%, the solubility decreased and protein aggregation was observed [76].

#### 3.3.2. Enzymes in Aqueous/Ionic Liquid Mixtures with Less than 50% IL

In this section work is described in which enzyme activity was supported in an aqueous phase modified by an ionic liquid.

Baeyer–Villiger monooxygenase performed benzyl ketone oxidation in aqueous buffer doped with miscible and non-miscible ionic liquids [77]. The ionic liquids with [PF_6_] anions appeared to be best systems, employing [BMP][PF_6_] at 30% IL led to >99% conversion to product in 2 h. No conversion was observed for [BMIM][MeSO_4_] at 30% IL but at 10% IL > 99% product formed at 4 h. [PF_6_] ionic liquids tend to be more organic soluble than water soluble and the successful ILs increased the substrate concentration in the reaction medium. This IL is not recommended for further use due to concerns over the stability of the [PF_6_] anion.

Immobilized lipase from *Candida antarctica* (Novozyme 435) exhibited an increase in glyceride hydrolysis in 30% alkyl imidazolium ILs of different anions [78]. The ILs with the hydrophobic anion [NTf_2_] and longer alkyl chains on the cation lead to increased activity.

The ability of lipase from *Burkholderia cepacia* to catalyse olive oil hydrolysis was measured in aqueous buffer mixed with phosphonium ILs (0.011–0.055 mol/L) with different anions [79]. Enhanced relative activities of 144 and 162 were observed in [P_6,6,6,14_][Phosp] and [P_6,6,6,14_][NTf_2_] at 0.055 mol/L concentration. These phosphonium ions confer significant hydrophobicity. Increasing the alkyl chain length decreased the activity observed for the phosphonium IL containing the chloride ion. Hydrophobic phosphonium ILs appear to be very promising additives for reactions involving greasy, more organic, substrates.

At elevated temperatures of 30–45 °C urate oxidase exhibited better activity in the oxidation of uric acid to allantoin in aqueous buffer containing 1% *v/v* triethanolammonium butyrate [TEAB], compared to ionic liquid-free conditions [80]. However, the activity decreased with increased ionic liquid content.

Human serum albumin (HSA) remained stable and folded in the presence of [Ch][dhp] and choline bitartrate [Ch][Bit] at 50 mg/mL IL concentration [81]. In addition, the ILs protected and refolded the protein from denaturant dextran.

Enzyme catalysed processes have also been operated in a mixture of ionic liquids and organic solvents. Lipase from *Geobacillus thermocatenolatus* performed high temperature (120 °C) enantioselective hydrolysis of racemic 2-(butyryloxy)-2-phenylacetic to mandelic acid in ethanol/[BMIM][BF_4_] and ethanol/[EMIM][BF_4_] [82]. In ethanol/[BMIM][BF_4_] only (*R*-) mendelic acid was formed at 12 h, however, changing the IL mixture to ethanol/[EMIM][BF_4_] led to (*S*-) madelic acid formation after 2.5 h. Lipase- Novozym 435 exhibited higher esterification activity of esculin (a natural phenolic glycosides) with palmitic acid in binary organic-ionic liquid media then the individual ionic liquid and organic solvents [83]. In the 1:1 binary mixture of trioctylmethylammonium bis(tri-fluoromethylsulfonyl) amide [TOMA][NTf_2_] and hexane, 92% conversion was observed at 60 °C at 96 h, whereas the conversion was 44% and 30% in [TOMA][NTf_2_] and hexane, respectively. In addition, the catalytic turnover number and efficiency in the binary [TOMA][NTf_2_]-hexane were 6.3 and 55 times higher than that of the catalytic performance in neat n-butanol.

### 3.4. Coating Enzymes with Ionic Liquids

The coating of an isolated enzyme in an ionic liquid has proven to be a very efficient method to support enzyme activity and increase stability and recyclability. The enzyme can be pure or immobilized. The enhancements in stability can be remarkable.

**Figure 6:**
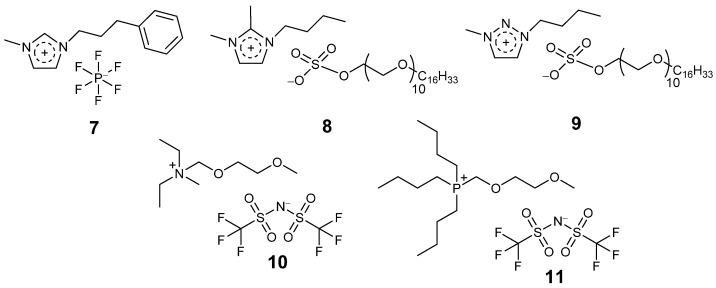
Structure of some ionic liquids (**7**–**11**) used for enzyme coating and storage.

Kim et al. first reported the coating of lipase from *Pseudomonas cepacia* in 1-(3′-phenylpropyl)-3-methylimidazolium) hexafluorophosphate [PPMIM][PF_6_] (**7**, Figure 6) for the transesterification of secondary alcohols in toluene at 25 °C [84]. This ionic liquid melts at 53 °C. The enzyme was coated by adding it as a powder to the IL in liquid form and mixing to prepare a homogeneous solution, before allowing it to solidify at room temperature. The IL coated enzymes were active and showed 1.5–2 times higher enantioselectivity compared to the native enzymes. In addition, the ionic liquid enzymes were recycled for six times maintaining 93% of activity of the native enzyme at the sixth run.

Itoh and co-workers have coated lipase from *Pseudomonas cepacia* within a surfactant containing imidazolium based ionic liquid [85,86]. The ionic liquid (**8**, Figure 6) coated lyophilized enzyme was used to catalyse the transesterification of 1-phenylethanol and vinyl acetate in diisopropyl ether, achieving 49% conversion in 2 h, compared to 15% conversion in 26 h for the free enzyme. The enhancement in the rate of transesterification was dependent on the substrate type, a 1100 times rate acceleration was observed for 1-(Naphthalen-2-yl)propanol with vinyl acetate in diisopropyl ether at 35 °C, whereas a 500 times rate acceleration was observed for 1-(Naphthalen-1-yl)ethanol. In addition, the ionic liquid coated enzyme was stable and retained full activity after storage in hexane for a week at room temperature, whereas the free enzyme lost activity completely. The immobilized enzyme was recycled for five times in the transesterification of 4-phenyl-3-butene-2-ol with vinyl acetate. The lyophilization and ionic liquid content were found to be critical for enzyme activity.

Itoh and co-workers entrapped lipase from *Burkholderia cepacia* in 1-butyl-3-methyl-1,2,3-triazolium cetyl-PEG10 sulphate ionic liquid (**9**, Figure 6) and investigated the transesterification of secondary alcohols [87,88]. The system was highly robust and the stabilized 9 coated enzyme in [N_2,2,1,MEM_][NTf_2_] ionic liquid (**10**, Figure 6) was stored for 2 years, with complete retention of activity. Alkyl-ether functionalized alkyl-phosphonium IL (**11**, Figure 6) was used as a solvent for IL 9 coated lipase mediated transesterification of secondary alcohols [89]. The IL **11** was recyclable and was reusable for more than 10 years.

### 3.5. Protein Modification to Manipulate Solubility in Ionic Liquids

**Figure 7:**
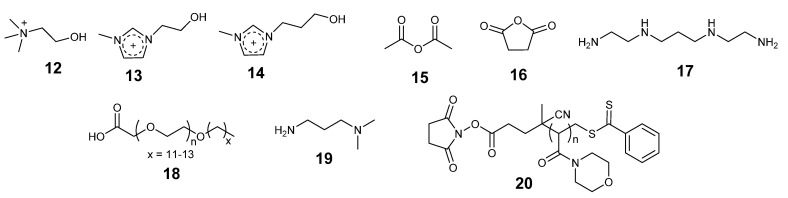
Chemical structure of some ionic liquid cations and modifiers (**12**–**20**) used to modify enzymes.

In the protein modification approach, the surface charges of the protein are modified to render them more soluble in the IL. Protein charge modification can be achieved by covalently bonding charged or neutral molecules. The hydrophilicity/hydrophobicity of the groups attached is also an important variable. The structures of some modifying groups are given in Figure 7. As an alternative to covalent modification, genetic manipulation by site-directed mutagenesis can also modify the protein surface.

Bekhouche et al. covalently modified lysine residues of formate dehydrogenase from *Candida boidinii* with carbonyldiimidazole activated ionic liquid cations cholinium (**12**, Figure 7), hydroxyethyl-methylimidazolium (**13**, Figure 7), and hydroxy-propyl-methylimidazolium (**14**, Figure 7) to increase enzyme solubility and prevent aggregation in high concentrations of [MMIM][Me_2_PO_4_] [90]. The modified enzyme retained 33–43% activity in the reduction of NAD^+^ to NADH using sodium formate as ion source in 70% (*v*/*v*) [MMIM][Me_2_PO_4_], at the same conditions the unmodified enzyme was inactive. The activity depended on the ionic liquid concentration, increasing the IL concentration decreased the activity observed.

Capping the protein surface by acetylation using **15** (Figure 7) or succinylation employing **16** (Figure 7) lowers the positive to negative surface charge ratio. This was applied to chymotrypsin and lipase in order to stabilize these enzymes in aqueous 10% (*v*/*v*) [BMIM][Cl] [91,92]. The activity and stability were found to decrease with increasing IL concentration.

Brogan et al. employed **17** and **18** (Figure 7) to modify myoglobin and solubilize it in neat anhydrous [BMP][OTf] and [BMP][NTf_2_] [93]. The α-helical content of the modified myoglobin was 69% in [BMP][OTf], compared to 72% for aqueous native myoglobin. A larger drop in helix content was observed in [BMP][NTf_2_] (61%). The β-sheet content of the modified protein increased to 6% and 8% respectively, compared to 1% in the aqueous protein. Enhanced thermal stability was observed for the modified enzyme in both the ILs. **17** and **18** were subsequently used to modify Glucosidase (Glu). The modified protein exhibited 30 times higher hydrolysis activity converting cellobiose to glucose at 110 °C in [EMIM][EtSO_4_] compared with the activity at 50 °C [94].

**18** and **19** (Figure 7) were used to modify α-Chymotrypsin. The modification enhanced the solubility of the enzyme in neat protic and neat aprotic ionic liquids (N-methyl-2-pyrrolidonium trifluoromethanesulfonate; [NMP][OTf]), or (1-methyl-3-(4-sulfobutyl)-1H- imidazol-3-ium trifluoromethanesulfonate; [HO_3_S(CH_2_)_4_MIM][OTf]) respectively and retained a similar β-sheet structure to the native protein in aqueous solution. The unmodified enzyme was insoluble in the ILs [95].

Poly(4-acryloylmorpholine) (PAcMO) (**20**, Figure 7) modified lipase A from *Bacillus subtilis* was soluble in [BMIM][PF_6_] and exhibited high activity in the transesterification of para-Nitrophenyl butyrate (pNPB) with ethanol [96]. The solubility and activity increased with increasing PAcMO on the enzyme. PAcMO also protected the enzyme from aggregation.

Dotsenko et al. engineered enzyme endoglucanase II from *Penicillium verruculosum* via rational design site-directed mutagenesis of three amino acids A52K, E70S, and V150L from three different sites distant from the enzyme active site to increase stability in [BMIM][Cl] and enhance the thermal stability of the enzyme [97].

A comprehensive single site saturation mutagenesis study was carried out on lipase A from *Bacillus subtilis* on its 181 amino acids residues to generate a library of 3620 variants to investigate the influence of amino acid residues on the enzyme stability in [BMIM] ionic liquids with anions of [Cl^−^], [Br^−^], [I^−^], and [OTf^−^] [98]_._ Mutation in more than 50% of the amino acid positions resulted in improved variants. The best variants contained mutations with chemically distinct amino acids, i.e., aliphatic to aromatic, polar to nonpolar. This study highlights the potential of directed evolution in ionic liquid compatible enzyme design. Molecular dynamic simulation studies of the interaction of the ionic liquid with lipase A from *Bacillus subtilis* suggested that the enzyme was stable in these ionic liquids, however, [BMIM] cations interact with the enzyme via surface residues resulting in lower activity. Reduction of the ionic liquid–enzyme interaction by enzyme surface charge modification should be a viable approach to achieving higher enzyme activity [99].

### 3.6. Enzyme Recycling Using Ionic Liquids

The inclusion of ionic liquids in isolated enzyme biocatalysis can facilitate methods to recycle the enzyme and therefore render it more economical to use. The following sections detail some potential uses of ionic liquids in enzyme recycling.

#### 3.6.1. Ionic Liquids in Enzyme Entrapment

Enzyme entrapment is a method of immobilization that imprisons the biocatalyst within a solid material or matrix. This generally happens during a polymerization event which occurs in the presence of the enzyme. The overall material is a gel, comprising an enzyme containing liquid and a solid matrix. Ionic liquids have been shown to confer properties to gels that assist in their application in green and sustainable applications [100].

We have reviewed different entrapment methods and the application of ionic liquid gels to the immobilization of enzymes recently [101], and this section will provide a summary of the technique.

The main classes of gel material are inorganic oxide gels, organic polymer gels, and supramolecular gels (gel formation initiated by Low Molecular Weight Gelators) [102,103,104].

The majority of work on enzymes entrapped in ionic liquid gels has been performed on inorganic oxide gels, and specifically sol-gel prepared silica gels. In a typical entrapment procedure, a silica precursor, such as tetraethoxyorthosilicate (TEOS), the enzyme, buffer, and the ionic liquid are added together in the presence of a sol-gel catalyst, such as a mineral acid. The mixture will then proceed through sol and gel states and once gelled is aged to ensure the formation of a robust silica network. For an example of the experimental procedure see Lee et al. [105].

Commonly hydrophobic ionic liquids have been used, and typical benefits quoted include higher recyclability, higher thermal stability, and greater activity compared to the entrapped enzyme in undoped silica. The third benefit can be rationalized due to specific ionic liquids effects. In addition, ionic liquids have a templating effect on the gel formation and may prevent the formation of a closed matrix structure, which would significantly slow the diffusion of the substrate.

The potential to tune and optimize the environment of ionic liquid gels should lead to increased interest in enzymes entrapped in ionic liquid gels, and an expansion of investigations into the fields of polymer, supramolecular, and hybrid materials.

#### 3.6.2. Ionic Liquid Tethering for Flow Biocatalysis

Flow biocatalysis is an emerging technology for sustainable chemical manufacturing. The use of flow can lead to significant process intensification due to high energy and mass transfer [106,107,108,109]. Lozano and Reetz pioneered the introduction of ionic liquids in flow biocatalysis [110,111,112,113,114,115]. Tethering an ionic liquid to a solid material can facilitate continuous flow processes [116,117].

Polyurethane supported microbeads of cellulose acetate doped with ionic liquid [OMIM][BF_4_] coated *Candida antarctica* lipase B (CALB) enabled 96% conversion of vinyl butyrate to butyl butyrate in a biphasic continuous transesterification reaction continuously for 18 days [118]. The technology was further employed for the transesterification of racemic (*R*,*S*)-1-phenylethanol with vinyl butyrate in [BMIM][PF_6_], [OMIM][BF_4_], and [BMIM][CF_3_SO_3_]. Both batch and continuous flow process produced > 99% ee for *R*-ester, however, in the continuous process the activity increased by a factor of 215 [119].

CALB doped [BMIM][PF_6_] ionic liquid droplets in oil were used for the transesterification of 1-phenylethyl alcohol to 1-phenylethyl acetate in a continuous flow process. The process was online for 4000 h and retained 77% initial activity with 272 g of product/mg of enzyme without enzyme leaching [120]. In comparison to the batch process, a 25-fold enhancement in activity was observed for the continuous process.

Biphasic- aqueous buffer and hydrophobic ionic liquid ([BMIM][NTf_2_] and [BMIM][PF_6_]) mixtures were developed for the continuous flow biocatalysis of Amano lipase from *Pseudomonas fluorescens*. In the hydrolysis of para-Nitrophenyl acetate the continuous system in buffer/[BMIM][NTf_2_] achieved 1.3 times product formation in 0.5 h compared to the reaction in a batch reactor [121].

#### 3.6.3. Ionic Liquid Sponges

**Figure 8:**
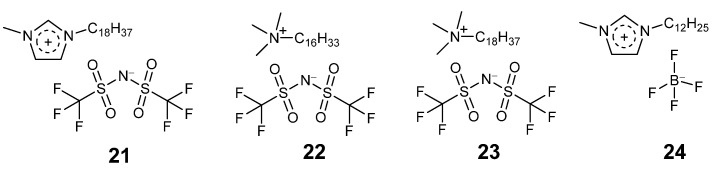
Chemical structure of sponge-like ionic liquids (**21**–**24**).

Lozano and co-workers have developed sponge-like long alkyl chain containing ionic liquids for chemical transformations and separation [44,122,123]. These long alkyl chain ionic liquids (for example, 1-methyl-3-octadecylimidazolium bis(trifluoromethylsulfonyl)amide (**21**, Figure 8)) are solid and sponge-like at room temperature (30 °C) and form a homogeneous solution at 50–60 °C in IL concentrations of higher than 30% *w/w* [124]. **21** was used to assist the transesterification of triacylglycerides with methanol using Novozym 435, a reaction that is important for biodiesel production. The reaction was conducted in the liquid phase IL at 60 °C. The reaction mixture was then cooled and centrifuged to separate the solid IL, the glycerol by-product, and the methyl ester product. The glycerol could be removed in water and the product could be removed in octane. The enzyme in ionic liquid could then be fully recovered and reused. A total of 96% product was generated in 6 h. The enzyme was recycled seven times without significant loss in activity.

Lipase in ionic liquid (**22**, Figure 8) catalysed the transesterification of aliphatic acids with alcohols to produce and separate sixteen different flavour esters with almost 100% product yield and seven recycles [125,126].

Lipase catalysed esterification of fatty acids and transesterification of vegetable oil were employed to produce oxygenated biofuels using ionic liquid (**23**, Figure 8). The IL enabled a cleaner biocatalytic reaction and product separation [127]. The product yield was more than 94% and the enzyme was recycled six times without loss of activity.

Esterification of fatty acids with glycerol in (**24**, Figure 8) gave 100% conversion to monoacylglycerides in 4 h with 100% selectivity and easy product separation [128].

## 4. Summary and Conclusions

Ionic liquids are unique fluids with a myriad of potential uses in biocatalysis. ILs have properties that can be tuned by systematic changes to the cations and/or anions and this gives them a competitive advantage over conventional molecular solvents. Of particular interest is the ability to alter the hydrophilicy from highly soluble in water, to completely immiscible.

Hydrophobic ionic liquids have potential applications in whole-cell bioprocesses as they can form a separate phase with aqueous buffer and therefore offer a less flammable and toxic alternative to organic solvents. ILs are uniquely useful solvents for this purpose as the dual properties of good polar molecule dissolution and high water immiscibility can be achieved. The real-life application of ionic liquids in whole-cell biocatalysis will depend upon the demonstration of ultra-low water solubility. This is because high volume fermentations require very large volumes of water and therefore the cost implications of polluting the aqueous phase could render the process uneconomical.

Hydrophobic and hydrophilic ionic liquids have been shown to affect proteins in isolated enzyme biocatalysis. In some cases interactions are detrimental, but increasingly protein–IL interactions are being discovered that support enzyme structure and activity. These beneficial interactions are finding applications improving the recycling, stability, and performance of isolated enzymes. The interactions of proteins with ILs can be probed by spectroscopy and understood on a molecular level, this, coupled with the tuneable nature of the ions in ILs, allows the systematic improvement of an isolated enzyme biocatalyst affording greater stability, activity, and recyclability.

All ionic liquids are different, and one must be careful when attempting to generalize about their properties. There are, however, universal barriers to the adoption of ionic liquids in industrial biocatalysis. The first is price; ionic liquids can be highly functionalized molecules, and this leads to costs that are significantly higher than for conventional solvents. The cost must therefore be minimized by working with ILs that have desirable properties, but do not require lengthy synthesis. The second is availability, as ‘designer’ solvents a specific IL is less likely to be available off the shelf. The third is water solubility and toxicity. As polar-like solvents ionic liquids, even the hydrophobic ILs could have some water solubility and this means that their effects on the environment must be assessed and monitored. All three of these barriers can be overcome by working with cheap, abundant ions derived from natural sources. Looking to the future ionic liquids applied in bioprocesses will be required to have high biocompatibility, low toxicity, and high recyclability. There is increasing interest in ionic liquids of this kind, and examples of classes of IL under investigation include amino acid, fatty acid and cholinium ILs [129,130,131,132,133].

Advances in genetics and molecular biology have enabled engineering of whole-cell biosynthetic pathways, and modification of enzymes by site-directed mutagenesis and directed evolution. These methods yield biocatalysts with broader substrate scopes, enhanced activity, and higher stability [14,134,135,136,137]. The interaction of these engineered biocatalysts with ionic liquid media is largely unstudied and presents exciting possibilities for the future.

As the art of ionic liquid design improves, we can expect a rapid adoption of ionic liquid technologies in biotechnology.
